# Assessment of the Clinical Interpreter of Death in Life-Threatening Infective Cases Admitted in the Intensive Care Unit of a North-Eastern State of India

**DOI:** 10.7759/cureus.13915

**Published:** 2021-03-16

**Authors:** Pollov Borah, Dilip K Saloi, Amarendra Deka, Rajib Hazarika, Ranjumoni Konwar, Putul Mahanta, Deepjyoti Kalita, Chiranjita Phukan, Kahua Das

**Affiliations:** 1 Anaesthesiology, Jorhat Medical College and Hospital, Jorhat, IND; 2 Anaesthesiology, Assam Medical College and Hospital, Dibrugarh, IND; 3 Radiology, Fakhruddin Ali Ahmed Medical College (FAAMC) and Hospital, Barpeta, IND; 4 Forensic Medicine and Toxicology, Assam Medical College and Hospital, Dibrugarh, IND; 5 Microbiology, All India Institute of Medical Sciences, Rishikesh, IND; 6 Internal Medicine, Tezpur Medical College and Hospital, Tezpur, IND; 7 Physiology, Tezpur Medical College and Hospital, Tezpur, IND

**Keywords:** sepsis, mortality, critical care centre, vital signs, severe sepsis

## Abstract

Objectives

The clinical factors affecting a patient's condition monitored over time could be useful not only to decide on an intervention that may increase the patients' possibilities of survival but also to predict the treatment outcome. Therefore, this study evaluates the clinical factors as predictors of mortality among severe sepsis patients admitted in the intensive care unit (ICU) of a tertiary care center.

Method

We did a prospective study on over 50 life-threatening infective cases with different causes admitted in the ICU. Clinical and biochemical parameters like temperature, heart rate, blood pressure, bicarbonate levels, blood lactate levels, and pH were monitored at admission, after 24 hours, and after 72 hours. The statistical analysis was done using Microsoft Excel (Microsoft Corporation, Redmond, WA) and the Statistical Package for the Social Studies (SPSS) version 22 (IBM Corp., Armonk, NY). We have obtained ethical clearance from the ethics committee (human) of Assam Medical College and Hospital, Dibrugarh. Before the collection of the data, we also took informed consent from the participants.

Results

The mean age of non-survivors was 44.35±11.64 years and that of survivors was 36.60±9.28 years, and the difference was statistically significant (p-value <0.003). An analysis of values of the various vital signs indicated substantial differences in the mean at different time intervals among survivors and non-survivors (p-value <0.05). Among non-survivors, mean temperature, pulse, and rate of respiration were observed to increase over time while blood pressure and oxygen saturation levels were significantly decreasing. Compared to survivors, the mean lactate levels of non-survivors at different time intervals were statistically significant (p-value <0.05). It is also observed that the pH of non-survivors was lower than survivors, and the mean pH value significantly different at different time intervals among the two groups (p-value <0.05).

Conclusion

The temperature, pulse, rate of respiration, blood pressure, and oxygen saturation levels are essential determinants of patient mortality in those suffering from a severe infection, besides serial lactate levels, bi-carbonate levels, and pH levels.

## Introduction

Life-threatening infective conditions are the foremost causes of morbidity and mortality globally, comprising 20% to 30% of deaths in the current clinical practice [1-2]. Infection in patients resulting in systemic inflammatory response syndrome (SIRS) [3] is associated with a rise in body temperature and heart and respiratory rate. It decreases the partial arterial pressure of carbon dioxide <32 mmHg, which predicts the fate of the individual’s infective status. Also, the condition of severe sepsis is allied with organ dysfunction. So, in septic shock, vasopressors need to maintain a mean arterial pressure of >65 mmHg, and the serum lactate level is required to be <2 mmol/l [4].

Therefore, these variables in censoriously ill patients predict the severity of the illness and death and evaluate the treatment costs. They also prescribe further required medical intervention besides screening the suitability and effectiveness of the ongoing course of remedial measures. A single clinical variable is unlikely to predict morbidity and mortality outcomes, hence the need for a group of clinical outcomes may come the closest in predicting morbidity and mortality for the patient suffering from sepsis [5].

The persistent rise of blood lactate level in the septicemic patient undergoing treatment in a critical care center (CCC) signifies hypoxic hypoxia and stagnant hypoxia and predicts death [5-7]. The delay of its clearance is observed with increased mortality. So, it is useful as a predictor of fatality and termination of therapy [8].

The current knowledge base for predicting morbidity and mortality following sepsis is still inadequate to implicate in this underdeveloped region of India. The conducted research, even if it exemplifies an essential aid for our knowledge of the issue of patients with severe sepsis, does not resolve the difficulties faced by us in determining the low-cost treatment while considering the affordability of saving the patient's life. Therefore, we evaluated the patients' clinical predictors, along with blood lactate levels, following septicemia and admission to the CCC.

## Materials and methods

A prospective study was carried out in the critical care center of the anesthesiology department. The study participants (n=50) were all major (>18 years), were suffering from sepsis, and have followed the natural course. Out of the cases studied, 20 suffered from respiratory failure, 15 from cardiovascular collapse, 10 from chronic renal failure, and five cases belonged to other categories. The heart rates, respiratory rate, urine output, and mean blood pressure were recorded hourly in the CCC during the course of treatment.

Vital parameters like oxygen saturation, partial pressure of oxygen (PO2), partial pressure of CO2 (pCO2), proton concentration (pH), bicarbonate ion (HCO3), and blood lactate level on admission, at 24 hours, and at 72 hours was monitored and recorded. Blood lactate level was analyzed by using the arterial blood gas (ABG) analyzer and was recorded. Each patient was monitored for four weeks to be registered as a survivor or non-survivor. The present study has followed the Surviving Sepsis Campaign: International Guidelines for Management of Sepsis and Septic Shock: 2018​ [9] guidelines to correct the sepsis markers described in the current study.

The patients were physically examined, and the data collected for statistical analysis, which was performed using the Statistical Package for the Social Sciences (SPSS) software version 22 (IBM Corp., Armonk, NY). Descriptive statistical methods were computed, and the student's t-test tested statistical significance. A p-value of less than 0.05 was considered statistically significant. We have obtained ethical clearance from the ethics committee (human) of Assam Medical College and Hospital (NO. AMC/EC/PG/100). Before collecting the data, we also took informed consent from the participants who could understand and sign the consenting form. In other cases, informed consent was obtained from the legal guardians.

## Results

**Figure 1:**
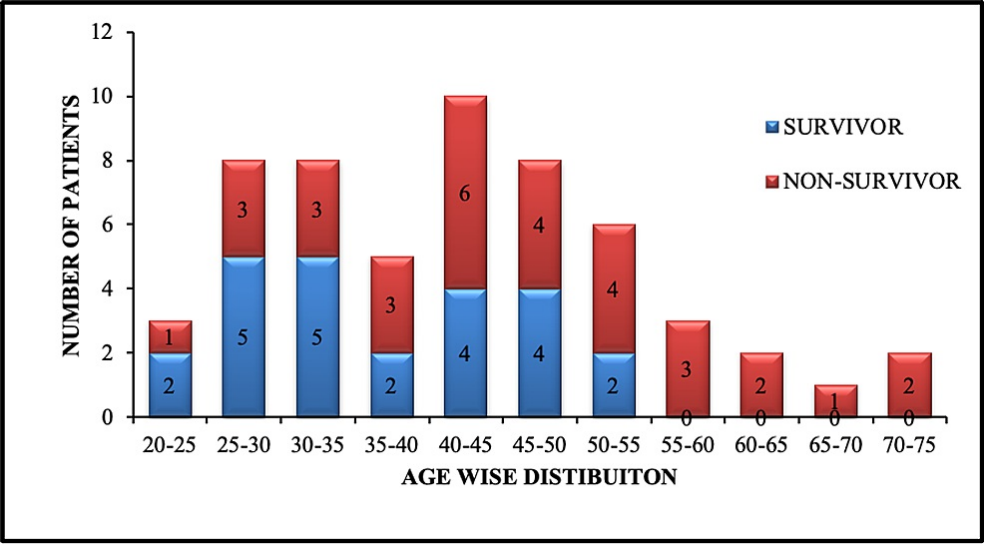
Age distribution among survivors and non-survivors

Out of 50 patients, 27 died during the four-week follow-up treatment, and 20% of the patients belonged to the age group of 45-50 years, as shown in Figure 1. The mean age of patients was 45.06±3.26 years. The mean (±) age of non-survivors was 44.35 (±11.64) years, and in survivors, it was 36.60 (±9.28) years. This result demonstrates the association of a higher age group with bad outcomes. The differences in mean age in the survivors and non-survivors groups were found to be statistically significant (p-value <0.003).

Changes in vital signs

Changes in vital signs monitored over time among the survivors and non-survivors groups are shown in Table 1. Significant differences in mean were observed in temperature, heart rate, rate of respiration, oxygen saturation levels between the two groups at the time of admission (p-value <0.05). Patients' mean body temperature was significantly different between the survivor and non-survivor groups at 24 hours and 72 hours with a gradual increase in temperature among non-survivors (100.530±2.5301) as compared to survivors (98.778±1.0505). A low level of mean blood pressure was observed among non-survivors as compared to survivors, and the difference was statistically significant at 24 hours and 72 hours. The mean blood pressure decreased from 66.07±7.820 to 50.78±8.679 in non-survivors. An increasing trend was observed in mean pulse and rate of respiration among non-survivors with increasing time. Those were significantly different from those of survivors at different time intervals (p-value < 0.05). Oxygen saturation was substantially lower among non-survivors with a mean SpO2 level of 92.19±9.540 at admission, 92.19±10.141 at 24 hours, and 91.70±10.622 at 72 hours while the mean SpO2 levels among survivors were > 95 at various time intervals. A significant decrease in urine output was also observed among non-survivors. The mean urine output among survivors and non-survivors was significantly different at 24 hours and 72 hours (p-value < 0.05).

**Table 1:** Vital signs of survivors and non-survivors over time

Vitals	At admission			24 Hours			72 Hours		
	Survivor	Non- survivor	p-value	Survivor	Non- survivor	p-value	Survivor	Non- survivor	p-value
Temperature	97.743± 1.1920	98.793± 1.2029	0.003	98.535± 1.0998	99.778± 1.8727	0.007	98.778± 1.0505	100.530± 2.5301	<0.001
Mean Arterial Pressure	71.52± 7.501	66.07± 7.820	0.16	73.70± 5.764	57.15± 6.938	<0.001	73.57± 7.668	50.78± 8.679	<0.001
Heart Rate	86.48± 9.638	95.74± 9.330	0.01	88.70± 13.492	116.3± 12.375	<0.001	89.30± 14.778	130.59± 16.230	<0.001
Respiratory Rate	22.52± 1.344	24.19± 1.688	<0.001	22.78± 1.858	26.41± 2.664	<0.001	23.22± 2.504	29.26± 3.748	<0.001
Oxygen Saturation (SpO_2_)	96.87± 2.341	92.19 ± 9.540	0.026	97.83± 1.435	92.19 ± 10.141	0.011	98.17± 1.466	91.70 ± 10.622	0.006
Urine Output	41.78± 14.666	44.00 ± 59.283	0.86	50.30± 17.867	27.59 ± 10.903	<0.001	46.91± 16.684	21.52 ± 12.274	<0.001

Lactate value among study participants

**Figure 2:**
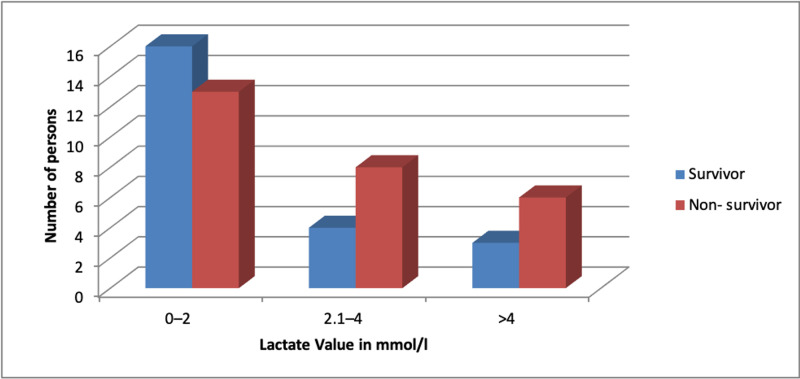
Distribution of study participants with different lactate values

Figure 2 represents the distribution of study participants with different lactate values. The lactate value in survivors ranged from 0.43 to 5.69 mmol/L while that in non-survivors ranged from 1.64 to 6.14 mmol/l. The majority of the non-survivors had a higher lactate value of >2 mmol/L. A significant change in patient numbers was observed at different lactate levels at different time intervals (p-value <0.05). Specifically, the number of patients with lactate value >4 mmol/L changed from 4 to 18 in 72 hours, as shown in Table 2.

**Table 2:** Distribution of study participants with different lactate values over time

Lactate Value (mmol/l)	On Admission	At 24 Hours	At 72 Hours	p-value
0-2	26	22	20	0.016
2.1-4	20	18	12	
>4	4	10	18	

Acid-base variables between the two groups

The changes in different acid-base variables were studied over time to standardize the impact on patient outcomes, as shown in Table 3. The t-test to test the mean difference revealed significant differences in mean bi-carbonate levels at admission, 24 hours, and 72 hours between survivors and non-survivors (p-value <0.01). In the case of lactate levels, it was observed that non-survivors had elevated mean lactate of >2 mmol/l at different time intervals, and compared to those among survivors, the difference in the lactate levels of non-survivors at different time intervals was statistically significant (p-value <0.05). It is also observed that the pH of non-survivors was lower than survivors, and the mean pH value was significantly different at different time intervals among the two groups (p-value <0.05). Reduced pH levels and increased lactate levels over time, thus indicate a rise in metabolic acidosis among patients resulting in a grave outcome.

**Table 3:** Changes in mean values of acid-base variables over time

Acid-Base variables	Outcome	Number (n)	MEAN ± S.D. (mmols/l)	p-value
Bicarbonate (HCO_3_)				
Admission	Not Survived	27	24.21 ± 8.312	0.003
	Survived	23	22.341 ± 8.211	
24 hours	Not Survived	27	20.211 ± 3.231	0.001
	Survived	23	13.34 ± 4.191	
72 hours	Not Survived	27	21.431 ± 6.314	0.001
	Survived	23	11.615 ± 3.214	
Lactate				
Admission	Not Survived	27	2.5204 ± 1.51498	0.000
	Survived	23	0.9545 ± 0.45798	
24 hours	Not Survived	27	2.5107 ± 1.63678	0.004
	Survived	23	1.2461 ± 1.21360	
72 hours	Not Survived	27	2.7904 ± 2.00160	0.023
	Survived	23	1.5496 ± 1.66788	
pH				
Admission	Not Survived	27	7.41 ± 0.02	0.001
	Survived	23	7.42 ± 0.09	
24 hours	Not Survived	27	7.23 ± 0.11	0.003
	Survived	23	7.36 ± 0.10	
72 hours	Not Survived	27	7.21 ± 0.14	0.001
	Survived	23	7.36 ± 0.11	

## Discussion

The scientific evaluation in 50 adult critically ill patients hospitalized in CCU had a reasonable mortality precision. Like a recent study, we found that the fatal group was of a substantially higher age [10], and the death is significantly (p<0.001) higher within the first 24 hours in the elders [11-12], as mentioned in some reviews.

In line with some previous studies, we found a gradual increase in temperature, low mean arterial pressure [13], an increasing trend in mean heart rate [14] and mean respiratory rate [15], lower SpO2, and a significant decrease in urinary output [16] was independently associated with the non-survivors. The above studies have reported similar clinical features among the non-survivors and survivors in a different time interval on admission, 24 hours, and 72 hours in-hospital stay. The significant difference found in oxygen saturation between the survivors and non-survivors groups of the present research was in agreement with a review [16].

The association of raised blood lactate with the state of tissue hypoxia is mentioned in a review in patients with overt circulatory failure. The higher lactate values in sepsis in non-survivors were consistent with some studies [11,15,17]. Another study revealed similar blood lactate levels in the non-survivors initially (3.1±2.3 mmol/l in non-survivors and 2.2±1.0 mmol/l in survivors) but had higher lactate levels after 12 hours (2.9±1.7 mmol/l in non-survivors and 1.6±0.9 mmol/l in survivors), after 24 hours (2.1±0.6 mmol/l in non-survivors and 1.5±0.7 mmol/l, in survivors), and after 48 hours (2.7±1.8 mmol/l in non-survivors and 1.9±1.4 mmol/l, in survivors) as compared with the survivors [18]. All these findings were in agreement with the present study.

Lactate clearance had a significant inverse relationship with mortality, as the decrease in blood lactate levels in the first 24 hours significantly give a good prognosis [13]. This study concurs with our research results, where we noticed that the mean arterial blood lactate range among non-survivors was increasing at the given intervals of time [19]. The high lactate level relating to high mortality found in the current study agrees with some reviews [16,20].

Like a previous review, we found decreased arterial bicarbonate concentration in the survivors' group after 24 and 72 hours [18]. Similar findings of arterial bicarbonate concentration in the non-survivors groups were noted, which increased after 72 hours as compared to 24 hours. The current findings were comparable with a review [13], where the pH results were 7.40±0.07 in survivors and 7.37±0.09 in non-survivors and the p-value was p<0.01.

Severity scores are essential assistants in managing sepsis patients in the CCC to predict the patient's outcome. It also helps in clinical decisions to find out a low-cost hospital stay. Some of the severity scales established for sepsis patients in the CCC during the last few decades are acute physiology and chronic health evaluation II (APACHE II), simplified acute physiology score (SAPS II), multiple organ dysfunction score (MODS), sequential organ failure assessment (SOFA), logistic organ dysfunction score (LODS), mortality prediction model II (MPM II) on admission, 24 hours, 48 hours, 72 hours, organ dysfunction and infection system (ODIN), three-day recalibrating ICU outcomes (TRIOS), and Glasgow coma score (GCS) [21].

APACHE II is the severity of the disease grading system developed in 1985 using the North American ICU patients' database [22]. The score is based on the physiologic measurement of the variable. The current study has used physiologic markers as mortality predictors, viz., temperature, blood pressure, heart rate, the rate of respiration, arterial pH, and SpO2 as mentioned in the above guideline. The SAPS II system was first described in 1992 to score the severity of the patient admitted to ICU. This system also includes physiologic variables besides age, admission type, and three-disease-related variables [23], and the current study partially used some of its variables. The MODS, an objective scale, has come up in 1995 to determine the severity of multiple organ dysfunction in sepsis patients [24]. However, the current study has not used this scoring system based on six organ failure scores. In 1994, the SOFA system was used by the European Society of Intensive Care Medicine, which was revised again in 1996. This qualified system of the severity of sepsis patient based on the degree of six organ malfunctions [25], which was not used in the current study. The LODS system was proposed primarily by Le Gall et al. in 1996 using 12 six organ failures [26]. The present study has not used this model to score predictive of the patient's outcome at ICU. MPM II, primarily described by Lemeshow et al. [27], evaluated the hospital death directly following sepsis at 24, 48, and 72 hours like that of the current study. The ODIN system was initiated by Fagon et al. This scoring system uses the recorded data within the first 24 hours at ICU admission to observe any malfunction of six organs, one infection, and the differentiates of prognosis [28]. In 2001, Timsit et al. [29] projected the TRIOS with daily SAPS II and LODS for sepsis patients admitted in the ICU for mortality prediction at 72 hours. The GCS is a worldwide tool for the fast calculation of an injured patient's consciousness level [30]. There was no sepsis case following injury; hence, we did not follow this mortality predictor.

Too many tasks determine the sepsis cases' hospital outcome for real-time decision-making for an effective and low-cost hospital stay. The clinical and biochemical predictors evaluated in the current study are convenient markers to determine in a set-up like ours, outreaching the updated technology to find out a low-cost management strategy.

Limitations of the study

The small sample size would have resulted in a less precise estimation of the frequency of different variables we studied. Besides, this study was conducted in a single-center, so the results may not be generalized to other centers explicitly dedicated to the management of sepsis patients. Also, the patients were followed up for four weeks. Hence, a more extensive multicentre study with a more extended period of patient follow-up may be useful.

## Conclusions

Changes in the vital clinical signs of patients suffering from sepsis were found at different time intervals during examination and management. Furthermore, the changes in acid-base variables during the course of admission could be useful determinants in predicting patient morbidity and mortality. Also, serial lactate levels, bi-carbonate levels, and pH levels may be significant clinical predictors of patient mortality.
